# Bilaterality as a Risk Factor for Recurrence in Papillary Thyroid Carcinoma

**DOI:** 10.3390/cancers15225414

**Published:** 2023-11-14

**Authors:** Hyeji Kim, Hyungju Kwon

**Affiliations:** Department of Surgery, Ewha Womans University Medical Center, 1071 Anyangcheon-ro, Yangcheon-Gu, Seoul 07985, Republic of Korea; rlagpwl1003@gmail.com

**Keywords:** papillary thyroid carcinoma, multifocality, bilaterality, recurrence

## Abstract

**Simple Summary:**

About 30% of thyroid cancer is found on both thyroid lobes simultaneously. However, the prognostic effect of bilaterality remains unestablished and controversial. This study of 1258 patients demonstrated that patients with bilaterality were associated with a more aggressive disease. We also found that patients with bilateral thyroid cancer showed higher recurrence rates than those with unilateral tumors. Therefore, patients with bilateral thyroid cancer might require careful treatment and follow-up approaches.

**Abstract:**

Previous studies suggested that the multifocality of papillary thyroid carcinoma (PTC) would increase the risk of recurrence; however, the impact of its bilaterality remains unclear. Between 2011 and 2018, 1258 patients with PTC underwent total thyroidectomy at Ewha University Medical Center. The 5-year recurrence-free survival rate was 95.7% in patients with bilateral PTC, while those with unilateral multifocal PTC and a unifocal tumor showed a 5-year event-free survival rate of 97.0% and 97.8%, respectively (*p* = 0.004). A multivariable Cox proportional hazards model indicated that bilaterality (HR 2.550, 95% CI 1.354–4.800), male sex (HR 2.010, 95% CI 1.007–4.013), and tumor size (HR 1.748, 95% CI 1.316–2.323) were associated with recurrence, although unilateral multifocality did not increase the risk of recurrence (HR 1.211, 95% CI 0.348–4.213). In conclusion, bilaterality was associated with aggressive features, including tumor size and microscopic ETE. Moreover, bilaterality was an independent predictor of recurrence in patients with PTC. Patients with bilateral PTC might require careful treatment and follow-up approaches.

## 1. Introduction

The incidence of thyroid cancer has risen continuously since the early 1980s, and now it is the ninth most frequent malignancy worldwide [1]. In 2020, there were 29,180 patients with newly diagnosed thyroid cancer in Korea, accounting for 11.8% of all cancer cases [2]. Papillary thyroid carcinoma (PTC) comprises 89.1% of thyroid cancer and usually has an excellent outcome. However, up to 35% of patients experience significant disease progression, such as regional recurrence or distant metastasis of thyroid cancer [3,4,5]. Numerous studies have tried to distinguish these higher-risk patients from the population with favorable prognosis [6,7]. Clinicopathological features, including multifocality, have been evaluated to predict recurrence or mortality.

Multifocality is defined as the presence of two or more distinct tumor foci within the thyroid gland, which have a prevalence rate of 18–87% [8]. Multifocality can be further divided into two groups: bilaterality and unilateral multifocality. Bilaterality in thyroid cancer is defined as malignancies diagnosed in both thyroid lobes simultaneously. The prevalence of bilateral PTCs ranges from 13% to 65%, which accounts for the majority of multifocal disease [9,10,11]. Recent meta-analyses revealed that multifocality could increase the risk of recurrence in patients with PTC; however, multifocal PTC might show distinctive characteristics according to its bilaterality, thus resulting in different rates of recurrence [12].

Several studies have investigated the prognostic implication of multifocality and its bilaterality in patients with PTC. Qu et al. reported that patients with bilateral PTC were associated with an increased risk of recurrence [13]. Wang et al. further showed that patients with bilateral PTC had shorter disease-free survival than those with unilateral multifocality [14]. Conversely, Cai et al. suggested that unilateral multifocal tumors were more likely to develop neck metastasis than bilateral PTCs [15]. Other researchers indicated that neither bilaterality nor unilateral multifocality was an independent predictive factor for poor outcomes in papillary thyroid microcarcinoma [16].

Therefore, in the present study, we evaluated the prognostic impact of bilaterality in patients with PTC.

## 2. Materials and Methods

### 2.1. Study Design

Our retrospective cohort study was approved by the Ewha University Mokdong Hospital Institutional Review Board (Approval No. 2022-07-032). The documentation of informed consent was waived.

### 2.2. Study Subjects

A total of 1258 patients with PTC were included in the present study who underwent total thyroidectomy from 2011 to 2018. Ultrasonography and computed tomography of the neck were performed preoperatively in all patients to evaluate tumor location, multifocality with its bilaterality, and lymph node (LN) metastasis. Patients with LN metastasis underwent LN dissection in addition to the conventional total thyroidectomy.

Clinicopathological data pertaining to patient demographics and tumor characteristics were reviewed. Primary tumor size, extrathyroidal extension (ETE), resection margin status, coexisting Hashimoto thyroiditis, LN metastasis, and multifocality with its bilaterality were identified and collected. Patients with incomplete or missing data were excluded.

Primary outcomes of the present study were recurrence rate and 5-year recurrence-free survival (RFS).

### 2.3. Definition of Clinicopathological Variables

The 8th American Joint Committee on Cancer tumor-node-metastasis (TNM) classification was used for pathologic staging. Tumor subtype was categorized according to the World Health Organization’s criteria for PTC variants. Recurrences were defined as newly detected malignant lesions on the thyroidectomy bed, logo-regional LNs, or distant metastases at least 1 year after initial surgery [17].

### 2.4. Radioactive Iodine (RAI) Ablation Protocol

Postoperative RAI ablation was conducted using ^131^I in selected patients, according to the risk stratification system of the American Thyroid Association [18]. A diagnostic whole-body scan was not routinely performed. In general, patients with intermediate risk of recurrence were recommended 1.1 GBq of RAI. For patients with aggressive pathologic features, including gross invasion and lateral neck node metastasis, higher activities (ranges of 3.7–5.6 GBq) of RAI were recommended. Coexisting comorbidities or patient’s preference were also considered for the final decision of administered RAI dose.

Six to twelve months after the first RAI treatment, stimulated thyroglobulin levels and antithyroglobulin antibody (TgAb) levels were obtained. For patients with positive TgAb, a subsequent diagnostic whole-body scan using 111 MBq ^131^I or ^123^I was also performed. In cases of failed ablation, a second dose of RAI (ranges of 1.1–5.6 GBq) was administered.

### 2.5. Follow-Up Protocol

The follow-up evaluations in patients with or without RAI treatment were conducted according to the ATA guideline [18]. Briefly, physical examination, measurement of serum thyroglobulin, and TgAb levels were performed every 6–12 months, and neck ultrasonography was performed annually. Levothyroxine was administered to maintain proper serum TSH levels, according to the ATA risk stratification [18].

### 2.6. Statistical Analysis

All statistical analyses were performed using SPSS Statistics 26.0 (IBM Corp., Armonk, NY, USA) and R 4.3.1 (R Development Core Team, Vienna, Austria). Dichotomous variable was compared using χ^2^ tests. Student’s *t*-test was used to compare continuous variables.

To reduce potential bias from confounders, including tumor size, ETE, resection margin involvement, and LN metastasis, 1:1:1 propensity score matching was performed. The 5-year RFS was estimated using Kaplan–Meier curves and log-rank tests.

Multivariable Cox proportional hazards regression model was used to identify predictive factors of recurrence. The proportional hazards assumption was graphically assessed and tested by the scaled Schoenfeld residuals; *p*-values < 0.05 were considered statistically significant.

## 3. Results

### 3.1. Patient Characteristics

The baseline clinicopathological characteristics of the included patients are described in Table 1. The follow-up period was 7.2 ± 3.4 years (interquartile range (IQR): 5.0–9.4 years). The mean age was 47.5 ± 11.5 years (IQR: 39.8–55.0 years), and 1100 patients (87.4%) were female. A total of 372 (29.6%) patients had bilateral PTCs, whereas 114 (9.1%) patients had unilateral multifocal tumors and 772 (61.1%) had unifocal tumors. Primary tumor size was 1.0 ± 0.7 cm with a median diameter of 1.1 cm (IQR, 0.6–1.2). Recurrences occurred in 43 patients (3.4%).

### 3.2. Recurrence Rates Comparison According to the Focality before Matching

**Figure 1:**
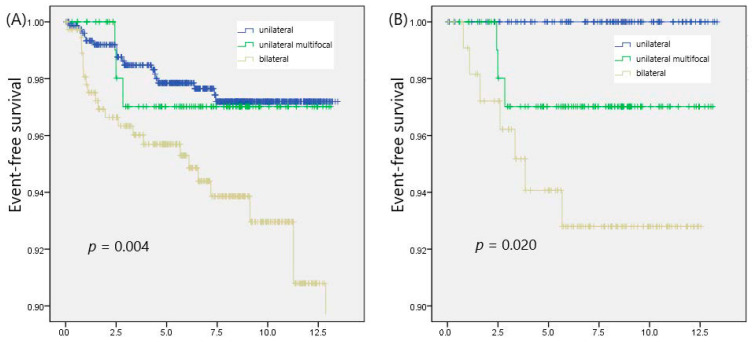
Event-free survival according to the focality of tumor in patients with PTC; (**A**) before and (**B**) after 1:1:1 matching.

Clinicopathological characteristics of the patients were compared according to the focality (Table 2). Bilaterality was associated with an increased tumor size (*p* = 0.010) and microscopic ETE (*p* = 0.001). LN metastasis (*p* = 0.031) showed a significant difference among groups. Recurrence was found in 22 (5.9%) patients in the bilaterality group, whereas 3 patients (2.6%) in the unilateral multifocality group and 18 (2.3%) in the unifocality group experienced recurrence (*p* = 0.007). The 5-year RFS was 95.7% in the bilaterality group and 97.0% in the unilateral multifocality group, respectively, while the unifocality group showed a 5-year RFS of 97.8% (*p* = 0.004; Figure 1A).

### 3.3. Recurrence Rates Cvmparison According to the Focaltiy after 1:1:1 Matching

After 1:1:1 propensity score matching, a comparison of characteristics among groups is shown in Table 3. Clinicopathological characteristics, such as tumor size, microscopic ETE, resection margin involvement, and LN metastasis, were comparable among groups. The recurrence rates (*p* = 0.022) and 5-year RFS (*p* = 0.022) across the groups still demonstrated significant differences after 1:1:1 matching (Figure 1B).

### 3.4. Identifying Risk Factors of Recurrence

Cox proportional hazards analysis was used to determine predictive factors of recurrence (shown in Table 4). Bilaterality (HR 2.446, 95% CI 1.297–4.616), male sex (HR 2.184, 95% CI 1.106–4.310), and tumor size (HR 1.703, 95% CI 1.305–2.223) were found to be independent risk factors of recurrence, while unilateral multifocality did not increase the risk of recurrence (HR 1.147, 95% CI 0.330–3.978).

## 4. Discussion

The present study demonstrated that bilaterality was associated with a higher risk of recurrence in patients with PTC. There is a controversy over whether unilateral multifocality and bilaterality are distinct multifocal entities [15]. Molecular and histopathologic analysis, including BRAF, RAS, and RET mutation status, indicated that up to 60% of multifocal PTCs might arise from different origins. Furthermore, tumors from separate clones are usually located in different thyroid lobes, while multifocal tumors within the same lobe tend to have the same mutational profile [19]. Wang et al. also suggested that bilaterality was an important character of PTC biology, which might enhance the clinician’s ability to predict the behavior of thyroid cancer [20].

Preoperative knowledge of the presence of bilateral disease is most relevant for decision-making in the management of PTC. Several researchers have tried to elucidate the predictive factors for bilaterality, because contralateral occult PTC could occasionally be found with an incidence of 12–40% [14,21]. A recent meta-analysis indicated that a tumor size over 1 cm, ipsilateral multifocality, or contralateral benign nodule was significantly associated with bilateral PTC [22]. Others further showed that total tumor size and number of tumor foci were predictive for bilateral involvement in PTC [23]. Tang et al. also suggested that patients with Hashimoto thyroiditis had a higher risk of bilateral PTC [24]. Our results were also comparable to those from previous studies.

Bilaterality has been considered a marker of extensive disease [16]. Recent guidelines and consensus statements recommended thyroid lobectomy in the majority of patients with PTC, although total thyroidectomy should be performed for bilateral thyroid cancer [18,25]. Previous reports indicated that bilaterality was associated with aggressive clinicopathologic features, including a larger tumor diameter, ETE, and LN metastasis, as well as a higher TNM stage at presentation [14,26]. In contrast, Kim et al. indicated that the clinicopathologic characteristics were not significantly different between the patients with multifocal–bilateral and multifocal–unilateral PTC [12]. In the present study, we demonstrated that bilaterality was associated with larger tumor size and microscopic ETE, which was consistent with the former ones.

There are conflicting views on whether bilaterality is an independent predictor for disease-free or overall survival in patients with PTC. Ding et al. showed that bilaterality could increase the risk of recurrence by 1.6 times [27]. Other researchers further indicated that bilaterality was a risk factor for neck recurrence, distant metastasis, or cancer-specific death [13,28]. Conversely, Polat et al. showed that bilateral multifocality was not associated with poor outcomes regarding loco-regional recurrences or distant metastases [29]. Yan et al. also reported that the recurrence-free survival was comparable between unifocal and bilateral PTCs after a follow-up period of 60 months [16]. Our result indicated that 5-year RFS was shorter in patients with bilateral tumors compared to those with unilateral multifocal or unifocal PTCs, after adjusting possible risk factors.

Our study has several limitations. First, the retrospective nature of the present study could be prone to selection bias. Bilaterality can be affected by various factors, including obesity, but we could not investigate these factors because of a lack of data [30]. Second, we did not evaluate long-term prognoses, such as mortality. A follow-up period of 7.2 years may not be enough for investigating cancer-specific survival. Third, we only investigated total thyroidectomy as the surgical procedure, while analyses of near-total or subtotal thyroidectomy might enhance our understanding. Surgical outcomes, including complications or recurrence, can vary according to the procedures [31,32]. Further studies with longer follow-up are required to address these issues. Finally, most of the patients enrolled in this study had the classic subtype of PTC. Other variants, including the tall cell or columnar cell variant, may affect the risk of recurrence. Further validation for the effect of bilaterality is warranted in other subtypes of PTC.

## 5. Conclusions

Bilaterality in patients with PTC was a predictive factor of recurrence. Bilaterality was further associated with aggressive features, including tumor size and microscopic ETE. Therefore, patients with bilateral PTCs might require careful treatment and follow-up approaches.

## Figures and Tables

**Table 1:** Patient demographics.

Characteristics	*n* = 1258
Age (years)	47.5 ± 11.5 (IQR: 39.8–55.0)
Female sex	1100 (87.4%)
Pathologic features	
Subtype	
Classical	986 (78.4%)
Follicular	249 (19.8%)
Tall cell	16 (1.3%)
Oncocytic	3 (0.2%)
Diffuse sclerosing	2 (0.2%)
Hobnail	1 (0.1%)
Columnar	1 (0.1%)
Primary tumor size (cm)	1.0 ± 0.7 (IQR: 0.6–1.2)
Microscopic ETE	769 (61.1%)
Focality	
Unifocality	772 (61.4%)
Unilateral multifocality	114 (9.1%)
Bilaterality	372 (29.6%)
Margin involvement	40 (3.2%)
Coexisting HT	347 (27.6%)
LN metastasis	
N0	728 (57.9%)
N1a	413 (32.8%)
N1b	117 (9.3%)
Postoperative treatment	
^131^I remnant ablation	558 (44.4%)
^131^I dose (mCi)	134.7 ± 34.5 (IQR: 100.0–150.0)
Recurrence	43 (3.4%)
Follow-up period (years)	7.2 ± 3.4 (IQR: 5.0–9.4)

ETE, extrathyroidal extension; HT, Hashimoto thyroiditis; LN, lymph node.

**Table 2:** Comparison of clinicopathological characteristics according to the tumor focality among patients with PTC.

Characteristics	Bilaterality (*n* = 372)	Unilateral Multifocal (*n* = 114)	Unifocal (*n* = 772)	*p*-Value
Age (years)	48.0 ± 11.3	48.0 ± 11.7	47.1 ± 11.5	0.345
Female sex	326 (87.6%)	95 (83.3%)	679 (88.0%)	0.377
Pathologic features				
Subtype				0.079
Classical	272 (73.1%)	92 (80.7%)	622 (80.6%)	
Follicular	95 (25.5%)	22 (19.3%)	132 (17.1%)	
Tall cell	3 (0.8%)	0 (0.0%)	13 (1.7%)	
Oncocytic	0 (0.0%)	0 (0.0%)	3 (0.4%)	
Diffuse sclerosing	1 (0.3%)	0 (0.0%)	1 (0.1%)	
Hobnail	1 (0.3%)	0 (0.0%)	0 (0.0%)	
Columnar	0 (0.0%)	0 (0.0%)	1 (0.1%)	
Primary tumor size (cm)	1.1 ± 0.7	0.9 ± 0.4	1.0 ± 0.7	0.010
Microscopic ETE	254 (68.3%)	74 (64.9%)	441 (57.1%)	0.001
Margin involvement	15 (4.0%)	6 (5.3%)	19 (2.5%)	0.151
Coexisting HT	103 (27.7%)	25 (21.9%)	219 (28.4%)	0.356
LN metastasis				0.031
N0	200 (53.8%)	58 (50.9%)	470 (60.9%)	
N1a	127 (34.1%)	46 (40.4%)	240 (31.1%)	
N1b	45 (12.1%)	10 (8.8%)	62 (8.0%)	
Postoperative treatment				
^131^I remnant ablation	178 (47.8%)	56 (49.1%)	324 (42.0%)	0.097
^131^I dose (mCi)	136.4 ± 40.1	131.4 ± 30.0	134.3 ± 32.0	0.622
Recurrence	22 (5.9%)	3 (2.6%)	18 (2.3%)	0.007
Follow-up period (years)	6.9 ± 3.4	7.1 ± 3.3	7.4 ± 3.4	0.043

PTC, papillary thyroid carcinoma; ETE, extrathyroidal extension; HT, Hashimoto thyroiditis; LN, lymph node.

**Table 3:** Comparison of clinicopathological characteristics according to the tumor focality among patients with PTC after 1:1:1 propensity score matching.

Characteristics	Bilaterality (*n* = 114)	Unilateral Multifocal (*n* = 114)	Unifocal (*n* = 114)	*p*-Value
Age (years)	50.0 ± 11.6	48.0 ± 11.7	47.5 ± 11.8	0.235
Female sex	101 (88.6%)	95 (83.3%)	94 (82.5%)	0.377
Pathologic features				
Subtype				0.708
Classical	86 (75.4%)	92 (80.7%)	92 (80.7%)	
Follicular	27 (23.7%)	22 (19.3%)	21 (18.4%)	
Tall cell	1 (0.9%)	0 (0.0%)	1 (0.9%)	
Primary tumor size (cm)	0.9 ± 0.4	0.9 ± 0.4	0.8 ± 0.4	0.312
Microscopic ETE	77 (67.5%)	74 (64.9%)	73 (64.0%)	0.845
Margin involvement	2 (1.8%)	6 (5.3%)	5 (4.4%)	0.354
Coexisting HT	22 (19.3%)	25 (21.9%)	28 (24.6%)	0.631
LN metastasis				0.907
N0	56 (49.1%)	58 (50.9%)	63 (55.3%)	
N1a	48 (42.1%)	46 (40.4%)	41 (36.0%)	
N1b	10 (8.8%)	10 (8.8%)	10 (8.8%)	
Postoperative treatment				
^131^I remnant ablation	55 (48.2%)	56 (49.1%)	54 (47.4%)	0.965
^131^I dose (mCi)	135.0 ± 48.6	131.4 ± 30.0	136.1 ± 28.2	0.783
Recurrence	7 (6.1%)	3 (2.6%)	0 (0.0%)	0.022
Follow-up period (years)	6.9 ± 3.4	7.1 ± 3.3	7.7 ± 3.3	0.161

PTC, papillary thyroid carcinoma; ETE, extrathyroidal extension; HT, Hashimoto thyroiditis; LN, lymph node.

**Table 4:** Predictive factors of recurrence in papillary thyroid carcinoma.

Characteristics	Univariate Analysis		Multivariate Analysis	
	HR (95% CI)	*p*-Value	HR (95% CI)	*p*-Value
Age	0.984 (0.957–1.012)	0.261	0.994 (0.968–1.021)	0.694
Male sex	2.870 (1.472–5.596)	0.002	2.184 (1.106–4.310)	0.024
Tumor size	2.097 (1.685–2.608)	<0.001	1.703 (1.305–2.223)	<0.001
Microscopic ETE	2.464 (1.182–5.138)	0.016	1.397 (0.639–3.056)	0.402
Resection margin	2.350 (0.722–7.650)	0.156	1.714 (0.508–5.783)	0.385
Hashimoto thyroiditis	0.591 (0.274–1.275)	0.180	0.666 (0.304–1.460)	0.310
LN metastasis	N0 (Ref.)		N0 (Ref.)	
N1a	2.296 (1.115–4.728)	0.024	1.343 (0.577–3.126)	0.494
N1b	6.888 (3.191–14.87)	<0.001	2.516 (0.967–6.544)	0.059
RAI ablation	3.797 (1.910–7.550)	<0.001	1.692 (0.708–4.042)	0.237
Focality	Unifocality (Ref.)		Unifocality (Ref.)	
Unilateral multifocality	1.154 (0.340–3.919)	0.818	1.147 (0.330–3.978)	0.829
Bilaterality	2.687 (1.440–5.011)	0.002	2.446 (1.297–4.616)	0.006

HR, hazard ratio; CI, confidence interval; ETE, extrathyroidal extension; LN, lymph node; Ref, reference value; RAI, radioactive iodine.

## Data Availability

The data presented in this study are available upon request from the corresponding author. The data are not publicly available due to institutional policy.
